# Assessing the Impact of CALGB 9343 on Surgical Trends in Elderly-Women With Stage I ER+ Breast Cancer: A SEER-Based Analysis

**DOI:** 10.3389/fonc.2019.00621

**Published:** 2019-07-09

**Authors:** Jose G. Bazan, James L. Fisher, Ko Un Park, Elizabeth A. Marcus, Marisa A. Bittoni, Julia R. White

**Affiliations:** ^1^Department of Radiation Oncology, Arthur G. James Comprehensive Cancer Center, The Ohio State University, Columbus, OH, United States; ^2^Division of Clinical Epidemiology, College of Public Health, The Ohio State University, Columbus, OH, United States; ^3^Division of Surgical Oncology, Department of Surgery, Arthur G. James Comprehensive Cancer Center, The Ohio State University, Columbus, OH, United States; ^4^Division of Breast Oncology, Department of Surgery, John H. Stroger, Jr. Hospital, Chicago, IL, United States; ^5^Division of Thoracic Oncology, Arthur G. James Comprehensive Cancer Center, Columbus, OH, United States

**Keywords:** elderly, ER+, mastectomy, race, SEER

## Abstract

**Purpose:** Lumpectomy (L) and breast radiotherapy (RT) results in equivalent outcomes in comparison to mastectomy (M) for early-stage breast cancer (BC) based on randomized controlled trials (RCT). Since 2004, RCT support that L without RT yields equivalent survival and acceptable local-regional outcomes in women ≥70-years old with T1N0 hormone-sensitive (ER+) BC on endocrine therapy. Based on this, we hypothesized that M rates should decrease substantially after 2004 in this low-risk elderly population.

**Methods:** We used the Surveillance Epidemiology and End Results registry data to conduct this study. We included women with T1N0 ER+ BC from 2000 to 2014. We compared M rates in women diagnosed from 2000 to 2004 vs. 2005–2012 using the Chi-Square test. Logistic regression analyses was performed to examine demographic/clinical factors associated with mastectomy.

**Results:** 67,506 women met the study criteria. In elderly Stage I ER+ BC, the M rate decreased by 6.3%: 29.0% before 2004 to 22.7% after 2004 (*p* < 0.0001). M rates remained higher in elderly non-Hispanic black (NHB, 27.1%, *p* < 0.0001), non-Hispanic Asian-Pacific-Islander (NHAPI, 30.1%, *p* < 0.0001), and Hispanics (24.4%, *p* = 0.0004) vs. non-Hispanic White (NHW, 21.5%). Treatment in the modern cohort was associated with decreased odds of mastectomy (OR = 0.71, 95% CI 0.68-0.74, *p* < 0.0001) while NH-API race was associated with the highest increased odds of mastectomy (OR = 1.65, 95% 1.53-1.78, *p* < 0.0001). In the modern cohort specifically (2005–2014), Hispanic women (OR = 1.12, *p* = 0.014), NHB women (OR = 1.21, *p* < 0.0001), and NHAPI women (OR = 1.73, *p* < 0.0001) all had higher odds of undergoing mastectomy relative to NHW women after adjusting for all other patient and tumor related factors.

**Conclusions:** In elderly patients with stage I, ER+ BC, M rates have decreased modestly since 2004. These trends are driven mostly be decreases in the M rate in NHW women, but M rates remain ~25% in Hispanic, NHB, and NHAPI women. Further research is needed to identify why M, which is associated with higher cost and morbidity than L alone, has not changed substantially in elderly, low-risk BC.

## Introduction

Randomized controlled clinical trials have established that breast conservation with lumpectomy and breast radiotherapy yields equivalent cancer outcomes to mastectomy for women with early stage breast cancer (1–4). In women that are ≥70 years old with clinical stage I (T1N0) estrogen-receptor positive (ER+) breast cancer on endocrine therapy, the Cancer and Leukemia Group B (CALGB) 9343 clinical trial data initially published in 2004 increasingly supports that lumpectomy alone compared to lumpectomy and breast radiotherapy results in equivalent survival and acceptable cancer control (5, 6). In the early publication by Hughes et al. the 5-year loco-regional recurrence (LRR) rates were 4% in women treated without radiotherapy compared to 1% in women treated with radiotherapy (6). The PRIME-II trial, which included a slightly different patient population compared to CALGB 9343 trial (patients ≥65 years-old, T1 or T2 tumors <3 cm), has demonstrated similarly low rates of LRR at 5 years of 4.1% without radiation and 1.3% with radiation (7). The updated CALGB 9343 results with 12.6 years median follow-up demonstrated 10-year LRR rates of 10 vs. 2% without and with radiotherapy, respectively (5). In addition, there was no difference in the secondary endpoints of overall survival, distant metastases or mastectomy-free survival between the two treatment arms. The CALGB 9343 mature data support that lumpectomy and endocrine therapy is an acceptable approach for elderly women with stage I, ER+, HER2 negative breast cancer and this is now incorporated as an NCCN guideline (8).

Numerous publications have examined the impact of the CALGB 9343 trial results on the use of adjuvant radiation therapy using both the Surveillance Epidemiology and End Results (SEER) registry data as well as the National Cancer Database (9–12). These studies show that use of adjuvant radiation has modestly decreased over time in this patient population. However, no studies have examined the impact of the CALGB 9343 trial data on surgical trends in elderly women with stage I ER+ breast cancer. It is well documented that access, travel distance and treatment duration of radiation therapy have been associated with less breast conservation and more frequent use of mastectomy for early stage breast cancer (13). We therefore hypothesized that since CALGB 9343 demonstrated lumpectomy and endocrine therapy without radiation is acceptable then mastectomy would become significantly less prevalent for treatment of small, hormone-sensitive, low risk breast cancers in elderly women.

## Methods

We used the National Cancer Institute SEER database to conduct this retrospective study. The SEER database collects cancer incidence data from 18-registries, representing approximately 27.8% of the total United States population. We selected women 70 years or older treated from 2000 to 2014. Patients were included if all of the following criteria were met: ER+ (any PR status); T1 tumors (T1mic, T1a-c); N0 nodal status; first cancer diagnosis. We excluded the following patients: no surgery performed; patients treated with mastectomy that received radiation therapy; unknown race; unknown tumor grade; unknown tumor histology; unknown marital status.

Patient demographic data included: patient age (dichotomized as ≥80 years old vs. <80 years old); race and ethnicity (non-Hispanic white [NHW] vs. non-Hispanic black [NHB] vs. non-Hispanic Asian-Pacific Islander [NHAPI] vs. non-Hispanic American Indian [NHAI] vs. Hispanic [H]); marital status (single [never married] vs. married/partnered vs. divorced/separated vs. widowed); county education level (dichotomized by the median value of percent of population with a bachelor's degree or higher); county income (dichotomized by the median value of the median income). Clinical data included: Tumor size (>1 cm vs. ≤1 cm); tumor laterality (right vs. left); PR status (positive or negative); tumor grade (1–2 vs. 3), histology (infiltrating ductal carcinoma [IDC], infiltrating lobular carcinoma [ILC], and other). We collected the following treatment data using the breast-cancer specific codes: type of surgery (mastectomy vs. lumpectomy) and radiation use in women that received lumpectomy (yes vs. no).

The patient population was further dichotomized into an early cohort (treated from 2000 to 2004) and a modern cohort (treated from 2005 to 2014) based on the initial publication of the CALGB 9343 trial. Our primary endpoint was to compare the mastectomy rate in the modern vs. early cohort. We hypothesized that the mastectomy rate is significantly lower in the modern vs. early cohort using the Chi-Square test with *p* < 0.05 considered significant. Our secondary endpoint was to identify demographic and clinical factors associated with the use of mastectomy. The Chi-Square test was used to test for differences between the mastectomy and lumpectomy cohorts. All factors with *p* < 0.10 were entered into a multivariate logistic regression analysis to calculate the adjusted odds-ratio (OR) for each factor. Statistical analysis was carried out using SAS version 9.4 (Cary Institute, NC).

## Results

### Patient Cohort and Characteristics

**Figure 1:**
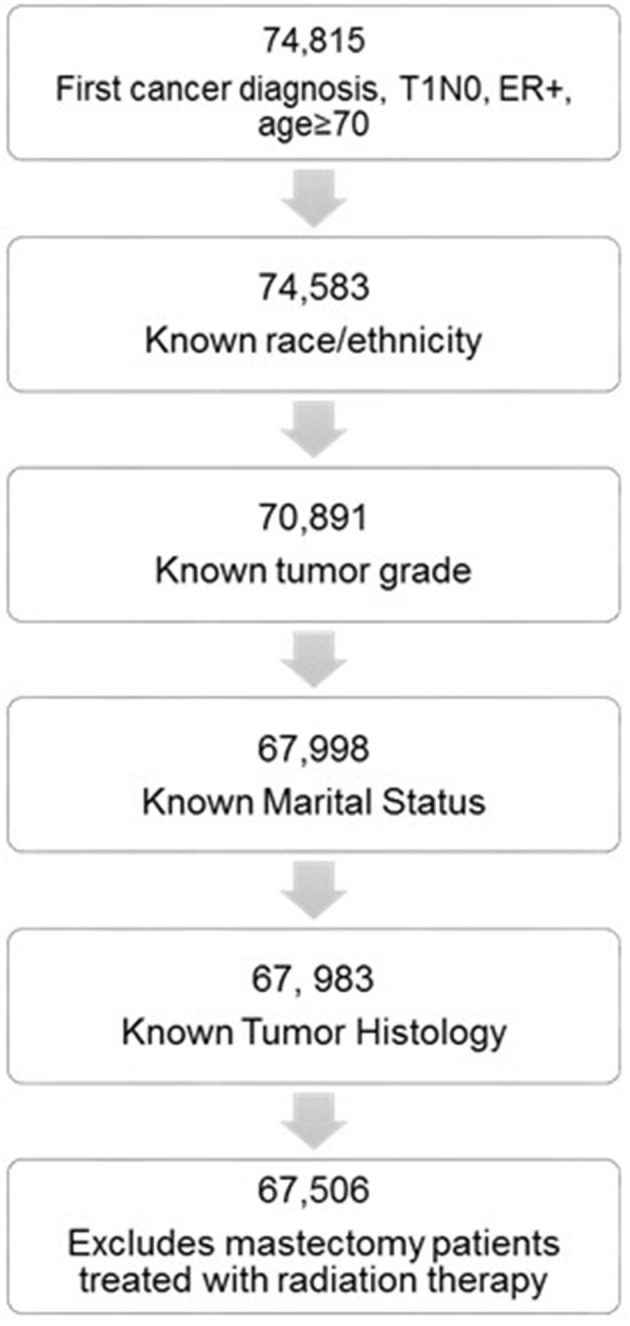
Flow diagram for analytic cohort.

We identified 67,506 elderly women with stage I ER+ breast cancer that met all the inclusion criteria (Figure 1). Of these 70.6% were treated in the modern era (2005–2014). Altogether, 50,964 women were treated with lumpectomy and 16,542 were treated with mastectomy. Baseline patient characteristics in the mastectomy and lumpectomy cohorts are shown in Table 1. With the exception of tumor laterality, there were imbalances in patient and tumor characteristics between the two groups. Women treated with mastectomy were more likely to be of non-white race/ethnicity, to be of older age, to come from counties with lower education and income levels, to have grade 3 tumors, and to have ILC histology. In the lumpectomy patients, 66.1% received radiation therapy, 32.1% did not receive radiation therapy, and delivery of RT was unknown in 1.8%. The rate of patients receiving adjuvant radiation therapy peaked at 72.1% in 2000 and declined to its lowest rate of 59.4% in 2014. Overall, the rate of patients receiving radiation did decline from 71.5% in the early cohort to 64.1% in the modern cohort (*p* < 0.0001).

**Table 1:** Patient characteristics by type of surgery.

	**Mastectomy** **(*N* = 16,542)**	**Lumpectomy** **(*N* = 50,964)**	***p***
Age			0.0047
≥80 years old	5,340 (32.3%)	15,854 (31.1%)	
70–79 years old	11,202 (67.7%)	35,110 (68.9%)	
Race and ethnicity			<0.0001
NHW	13,296 (80.4%)	42,592 (83.6%)	
NHB	1,058 (6.4%)	2,689 (5.3%)	
NHAPI	1,141 (6.9%)	2,560 (5.0%)	
NHAI	54 (0.3%)	153 (0.3%)	
H	993 (6.0%)	2,970 (5.8%)	
Marital status			<0.0001
Married	7,088 (42.9%)	23,783 (46.7%)	
Single (never married)	1234 (7.5%)	3,573 (7.0%)	
Divorced	1,327 (8.0%)	4,447 (8.7%)	
Widowed	6,893 (41.6%)	19,161 (37.6%)	
County education level			<0.0001
>24.9% bachelor's degree	7,001 (42.3%)	26,547 (52.1%)	
≤24.9% bachelor's degree	9,541 (57.7%)	24,417 (47.9%)	
County household income level			<0.0001
>$46,120	6,930 (41.9%)	26,885 (52.8%)	
≤$46,120	9,612 (58.1%)	24,079 (47.3%)	
Tumor laterality			0.3222
Right	8,075 (48.8%)	25,184 (49.4%)	
Left	8,466 (51.2%)	25,776 (50.6%)	
Tumor grade			<0.0001
2-Jan	13,994 (84.6%)	45,085 (88.5%)	
3	2,548 (15.4%)	5,879 (11.5%)	
Tumor size			<0.0001
>1cm	9,965 (60.2%)	25,882 (50.8%)	
≤1cm	6,577 (39.8%)	25,082(49.2%)	
Tumor histology			0.0004
IDC	13,699 (82.8%)	42,382 (83.2%)	
ILC	1,454 (8.8%)	4,036 (7.9%)	
Other	1,389 (8.4%)	4,546 (8.9%)	
Tumor PR status			<0.0001
PR+	13,953 (84.4%)	43,968 (86.3%)	
PR-	2,589 (15.6%)	6,996 (13.7%)	
Treatment era	<0.0001		
Early cohort (2000–2004)	5,755 (34.8%)	14,125 (27.7%)	
Modern cohort (2005–2014)	10,798 (65.2%)	36,839 (72.3%)	
Radiation therapy			N/A
Yes	0	16,347 (32.1%)	
No	16,437 (99.4%)	33,708 (66.1%)	
Unknown	105 (0.6%)	909 (1.8%)	

*NHW, non-hispanic White; NHB, non-hispanic Black; NHAPI, non-hispanic Asian-Pacific Islander; NHAI, non-Hispanic American Indian; H, Hispanic; IDC, infiltrating ductal carcinoma; ILC, infiltrating lobular carcinoma; PR, progesterone receptor; N/A, not applicable; Laterality unknown in 1 patient in the mastectomy group and 4 patients in the lumpectomy group*.

### Mastectomy Trends Over Time

**Figure 2:**
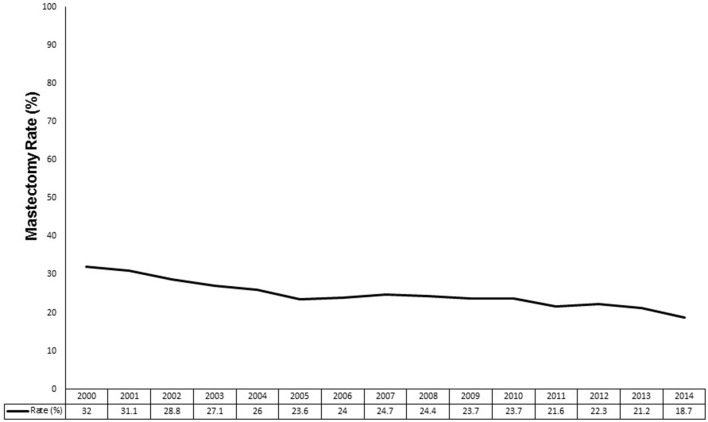
Mastectomy rates by year from 2000 to 2014.

Figure 2 demonstrates the mastectomy rate by year from 2000 to 2014. The mastectomy rate was at its highest at 32.0% in 2000 and decreased to a nadir of 18.7% in 2014. Overall, the mastectomy rate decreased from 29.0% in the early era to 22.7% in the modern era (*p* < 0.0001).

**Figure 3:**
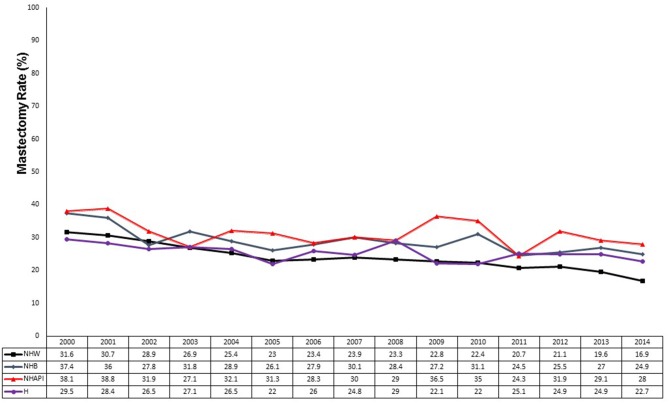
Mastectomy rates by race from 2000 to 2014. Black line, non-Hispanic Whites (NHW); blue line, non-Hispanic Blacks (NHB); red line, non-Hispanic Asian-Pacific Islanders (NHAPI); purple line, Hispanics.

We analyzed the surgical trends over time by race and ethnicity (Figure 3). The mastectomy rate decreased from 28.7 to 21.6% in NHW women (*p* < 0.0001). Compared to NHW women, NHB women started at a numerically higher rate of mastectomy in the early cohort (32.3 vs. 28.7%, *p* = 0.025). The mastectomy rate in NHB women significantly decreased to 27.1% (*p* = 0.003 compared to early cohort) but remained significantly higher compared to the 21.6% mastectomy rate in white women (*p* < 0.0001). Similarly, NHAPI women had a significantly higher mastectomy rate compared to white women in the early cohort (33.2 vs. 28.7%, *p* = 0.004) and in the modern cohort (30.1 vs. 21.6%, *p* < 0.0001). The absolute 3.1% decrease in mastectomy rate in NHAPI women in the modern era was not statistically significant (*p* = 0.080) and was a small absolute decrease compared to NHW women (7.1%) and NHB women (5.2%). Hispanic women started with a low baseline mastectomy rate (27.5%) that declined to 24.4% in the modern era (*p* = 0.061). While the mastectomy rate in Hispanic women compared to NHW women was not significantly different in the early cohort (27.5 vs. 28.7%, *p* = 0.447), the mastectomy rate in Hispanic women showed a smaller absolute decline resulting in a significantly higher mastectomy rate compared to NHW women in the modern cohort (24.4 vs. 21.6%, *p* = 0.0004). However, the mastectomy rate in NHW women in the modern cohort was significantly lower compared to NHB (*p* < 0.0001) and NHAPI (*p* < 0.0001) women in the modern cohort. There were a small number of NHAI women in both the early cohort (*N* = 49) and modern cohort (*N* = 158) with no statistically significant change in the mastectomy rate over time from 20.4% (10/49) to 27.9% (44/158), *p* = 0.300.

### Patient and Tumor Characteristics Associated With Mastectomy

Given that there were imbalances in patient and tumor characteristics between the mastectomy and lumpectomy cohorts in every factor except for tumor laterality, all remaining variables were entered into a multivariate logistic regression model. Table 2 demonstrates the results. Women treated in the modern era had a 29% reduction in the odds of receiving mastectomy (OR = 0.71, 95% CI 0.68-0.74, *p* < 0.0001). Women living in wealthier counties had a similar 28.4% reduction in the odds of undergoing mastectomy. Significant reductions in the odds of undergoing mastectomy were also seen for women living in counties with higher proportion of population with at least a bachelor's degree (OR = 0.85, *p* < 0.0001), and for women with PR+ tumors (OR = 0.90, *p* < 0.0001).

**Table 2:** Multivariate logistic regression analysis for predictors of receiving mastectomy in the entire cohort.

	**Odds ratio**	**95% CI**	***p***
Age			
≥80 years old	1.013	0.974–1.054	0.5094
70–79 years old	Reference	Reference	
Race/Ethnicity			
NHW	Reference	Reference	
NHB	1.182	1.096–1.275	<0.0001
NHAPI	1.651	1.534–1.778	<0.0001
NHAI	1.092	0.797–1.495	0.1851
H	1.063	0.985–1.146	0.1142
Marital status			
Married	Reference	Reference	
Single (never married)	1.158	1.079–1.244	<0.0001
Divorced	1.017	0.950–1.088	0.6298
Widowed	1.159	1.111–1.207	<0.0001
County education level			
>24.9% bachelor's degree	0.846	0.803–0.892	<0.0001
≤24.9% bachelor's degree	Reference	Reference	
County household income level			
>$46,120	0.716	0.679–0.755	<0.0001
≤$46,120	Reference	Reference	
Tumor grade			
2-Jan	Reference	Reference	
3	1.285	1.220–1.353	<0.0001
Tumor size			
>1cm	1.415	1.364–1.467	<0.0001
≤1cm	Reference	Reference	
Tumor histology			
IDC	Reference	Reference	
ILC	1.122	1.053-1.197	0.0004
Other	0.939	0.881-1.001	0.0545
Tumor PR status			
PR+	0.9	0.856–0.946	<0.0001
PR-	Reference	Reference	
Treatment era			
Early cohort (2000-2004)	Reference	Reference	
Modern cohort (2005-2014)	0.71	0.684–0.738	<0.0001

*NHW, non-Hispanic White; NHB, non-Hispanic Black; NHAPI, non-Hispanic Asian-Pacific Islander; NHAI, non-Hispanic American Indian; H, Hispanic; IDC, infiltrating ductal carcinoma; ILC, infiltrating lobular carcinoma; PR, progesterone receptor*.

Compared to NHW women, NHAPI women had a >1.6-fold increase in the odds of receiving a mastectomy (OR = 1.65, 95% 1.53-1.78, *p* < 0.0001). NHB race was also associated with higher odds of mastectomy (OR = 1.18, *p* = 0.0001). Relative to NHW women, Hispanic women did not have a significantly increased odds of undergoing mastectomy (OR = 1.06, *p* = 0.114). Pathologic characteristics associated with higher odds of receiving mastectomy included tumors >1 cm (OR = 1.42, *p* < 0.0001), grade 3 tumors (OR = 1.29, *p* < 0.0001) and women with ILC histology (OR = 1.12, *p* < 0.0001). Relative to married women, single women (OR = 1.16, *p* < 0.0001) and widowed women (OR = 1.16, *p* < 0.0001) had a higher odds of receiving mastectomy.

The factors not associated with either higher or lower odds of receiving mastectomy included age ≥ 80 years vs. <80 years (OR = 1.01, *p* = 0.509), NHAI race/ethnicity (OR = 1.09, *p* = 0.185), and divorced (relative to married) women (OR = 1.02, *p* = 0.630). Women with tumor histology other than IDC or ILC had a trend toward lower odds of receiving mastectomy (OR = 0.94, *p* = 0.055).

Given that we saw numerically smaller decreases in mastectomy rates over time in NHB, NHAPI, and Hispanic women compared to NHW women, we performed a final multivariate analysis of factors associated with mastectomy only in the modern cohort (Table 3). Relative to NHW women, Hispanic (OR = 1.12, *p* = 0.014, NHB (OR = 1.21, *p* < 0.0001) and NHAPI (OR = 1.73, *p* < 0.0001) women had significantly higher odds of undergoing mastectomy after adjusting for all other patient and tumor related factors.

**Table 3:** Multivariate logistic regression analysis for predictors of receiving mastectomy in the modern cohort.

	**Odds ratio**	**95% CI**	***p***
Age			
≥80 years old	0.979	0.932–1.027	0.3814
70–79 years old	Reference	Reference	
Race/Ethnicity			
NHW	Reference	Reference	
NHB	1.213	1.112–1.324	<0.0001
NHAPI	1.734	1.592–1.889	<0.0001
NHAI	1.308	0.920–1.859	0.1349
H	1.115	1.022–1.216	0.0144
Marital status			
Married	Reference	Reference	
Single (never married)	1.217	1.120–1.323	<0.0001
Divorced	1.028	0.950–1.113	0.4955
Widowed	1.14	1.085–1.198	<0.0001
County education level			
>24.9% bachelor's degree	0.816	0.765–0.870	<0.0001
≤24.9% bachelor's degree	Reference	Reference	
County household income level			
>$46,120	0.734	0.688–0.783	<0.0001
≤$46,120	Reference	Reference	
Tumor grade			
2-Jan	Reference	Reference	
3	1.334	1.251–1.422	<0.0001
Tumor size			
>1cm	1.369	1.309–1.431	<0.0001
≤1cm	Reference	Reference	
Tumor histology			
IDC	Reference	Reference	
ILC	1.156	1.072–1.247	0.0002
Other	1.01	0.932–1.094	0.8148
Tumor PR status			
PR+	0.918	0.861–0.978	0.0079
PR-	Reference	Reference	

*NHW, non-Hispanic White; NHB, non-Hispanic Black; NHAPI, non-Hispanic Asian-Pacific Islander; NHAI, non-Hispanic American Indian; H, Hispanic; IDC, infiltrating ductal carcinoma; ILC, infiltrating lobular carcinoma; PR, progesterone receptor*.

## Discussion

This study is the first to report the impact of the CALGB 9343 results on surgical trends for elderly women with stage I, lymph-node negative ER+ breast cancer. Consistent with our hypothesis, we found that the mastectomy rate declined modestly from the pre-publication era (2000–2004) to the post-publication era (2005–2014). However, we also found that disparities in the surgical management of this low-risk breast cancer population exist even in the modern cohort. Nearly 25% of Hispanic women, more than 25% of elderly NHB women and >30% of elderly NHAPI women underwent mastectomy from 2005 to 2014 compared to approximately one-fifth of NHW women. These racial/ethnic disparities persisted after adjusting for other patient- and tumor-related factors.

Given that lumpectomy and endocrine therapy without radiation therapy is recognized as an acceptable treatment for women ≥70 years old with stage I, ER+ breast cancer, we expected that mastectomy rates would decline in these women. There are potential benefits to reduction of mastectomy utilization in this population. While a safe operation, hospitalization and longer recovery periods are required after mastectomy vs. lumpectomy. Mastectomy without reconstruction can result in postural changes (14, 15), and for older patients, this may negatively influence balance.

Comprehensive analyses of local-regional treatment patterns (mastectomy vs. lumpectomy alone vs. lumpectomy+radiation) in elderly women with early-stage breast cancer are few. Lemasters et al. has performed the most detailed analysis using SEER-Medicare data from 2003 to 2009 (16). Comparisons with our study are limited as Lemasters et al. included women with higher stage breast cancer (stage II), ER- disease, and younger women aged 66–69 years old. Nonetheless, the mastectomy rate was 23.1%, which was highest in non-agenerians (30.9%) compared to women aged 66–69 years old (20.0%). The mastectomy rate was 15.6% in women with clinical stage I disease, and was 21.6% for patients with ER+ breast cancer. This study did not report changes in the mastectomy rate over the time period examined or by race/ethnicity.

In regards to stage I, node-negative breast cancer, at least two series have examined patterns of surgery in women of all ages as well as by ER status. In a SEER study by Showalter et al. the mastectomy rate in Stage I breast cancer (<60% ER+) declined significantly from 60% in 1988–1992 to 41% in 1993–1997 to 33% in 1998–2002 and finally to 32% from 2003 to 2007 (17). This demonstrates a relatively steady mastectomy rate over the interval from 1998 to 2007. Interestingly, age was not a factor associated with surgical treatment (17). Similarly, Vaz-Luis et al. examined mastectomy rates from 2000 to 2009 in 10,249 women with stage I, lymph node negative breast cancer (~80% ER+) from 9 academic institutions (18). In this study, the mastectomy rate was 23.9% and there was no change over time. The mastectomy rate was lowest in the cohort of 1,700 women aged ≥70 years-old at 17% (18). A higher rate of mastectomy (21.5%) was found in the more modern cohort (2005–2014) of elderly women in the SEER database in this study This is consistent with recent data that has demonstrated a rise in mastectomy rates in women with early-stage breast cancer since the early 2000s (19, 20). However, our study does demonstrate that in the select group of elderly low-risk breast cancer patients (women>70 years of age with stage I, ER+ disease) mastectomy rates have declined since publication of CALGB 9343 with an absolute decrease of 7.3%.

Significant variation in the surgical management of stage I, ER+ breast cancer in elderly women was identified based on patient race/ethnicity. In this study, we found that NHB, NHAPI, and Hispanic women had significantly higher mastectomy rates than white women, even after adjusting for the treatment era and other patient/tumor characteristics. Most notably, NHAPI women had >1.6-fold odds of receiving mastectomy compared to white women. Gelber et al. found that relative to white women, Japanese and Filipino women had significantly higher mastectomy rates in a cohort of >95% stage I-II breast cancer from the SEER-Hawaii registry (21). Prehn et al. examined the impact of API race on mastectomy rates in 1,772 women with localized breast cancer from the SEER-San Francisco Bay Area registry and found that the mastectomy rate was 58% for API women compared to 42% for white women (22). While not specific to the elderly stage I ER+ breast cancer population, these studies demonstrate disparities in the surgical management of API women with breast cancer favoring high rates of mastectomy.

Racial disparities in the outcomes from breast cancer are well documented with black women having consistently lower survival rates compared to white women (23–25). In addition, relative to white women, black women have been found to have more unfavorable tumor characteristics including younger age of diagnosis (26), larger tumor size (27, 28), higher rates of triple-negative disease (23), and higher rates of grade 3 tumors (29, 30). In our study, NHB women > 70 years of age are more likely to receive mastectomy compared to similar aged white women, even when looking only at the modern cohort. However, this cannot only be explained by a prevalence of unfavorable disease characteristics as all studied had low risk breast cancer (stage I, ER+).

Compared to NHB and NHAPI women, Hispanic women had lower mastectomy rates in both the early and modern cohorts. However, similar to NHB and NHAPI women, Hispanic women saw a small decline in mastectomy rates over time and remained with a significantly higher mastectomy rate relative to NHW women in the modern cohort. Therefore, the impact of the CALGB 9343 publication on decreasing mastectomy rates was not as pronounced in all minority groups relative to NHW women.

The underlying reasons for these disparities in surgical management are manifold and complex. Surgical choice is driven by patient concerns about recurrence, radiation, and body image (31). In elderly women with stage I, ER+ breast cancer contemplating lumpectomy, concerns about radiation should be lessened since omission of radiation is an acceptable option based on CALGB 9343. However, minority patients may not have access to or may choose not to seek out high-volume academic facilities, where it has been shown that the CALGB 9343 has had the most impact in terms of decreased radiation use (9). In a study by Freedman et al., Black and Hispanic patients were less likely to select their surgeon based on reputation relative to White women, and they were also less likely to select their hospital based on reputation (32). The power of culture cannot be underestimated—in a study by Maly et al., older Latina women were more likely to identify a family member as the final treatment decision-maker relative to African-American and white women (33). Further, patients were less likely to receive lumpectomy when the family made the final treatment decision (33). Therefore, interventions that promote education of the family decision-maker and interventions designed to empower minority patients to become more involved in choosing their treatment facility and physicians may have an impact in the future in terms of decreasing mastectomy rates for elderly women with stage I, ER+ breast cancer.

The impact of marital status on surgery type in older women with stage I breast cancer is also understudied and the reported data, which includes younger patients, are mixed. In women with stage I breast cancer (34% elderly), Showalter et al. found that single (never married) women and the combined group of divorced/widowed/separated women all had higher odds of mastectomy compared to married women. Al-Refaie et al. reported on factors associated with mastectomy in stage 0-II breast cancer (age not reported) at the University of Texas MD Anderson Cancer Center from 2002 to 2003 (34). In this cohort of 293 women, 31% underwent mastectomy. On multivariate analysis, the only factor associated with mastectomy was being a widow. In our analysis, we found that widows did not have a higher odds of mastectomy compared to married women whereas divorced women and single (never married) women had higher odds of undergoing mastectomy. Lastly, Lizarraga et al. examined factors associated with mastectomy in women with stage I-III breast cancer (51% stage I, 25% elderly, 84% ER+ or PR+) using the SEER 2010–2011 data (35). These authors found that relative to married women, single women had a lower odds of receiving mastectomy. Again, this contrasts the elderly stage I, ER+ SEER data from this study, in which single women had higher odds of mastectomy.

This study has several limitations. Due to the retrospective design, there are no data regarding how patient choice influenced receipt of mastectomy or lumpectomy. In addition, there are important clinical and pathologic variables—including tumor extent, tumor focality/centricity, surgical margin status, and patient germline mutations—that are unavailable in the SEER database but could potentially contribute to the surgical decision. Furthermore, HER2 status is only available from 2010-present, so a detailed analysis on the impact of HER2 status on type of surgery cannot be performed for the entire cohort. While it would be interesting to know if loco-regional failure (LRF) rates are different amongst the race/ethnic groups, LRF is not an endpoint captured in the SEER database. Even if LRF events were captured, the lack of the already mentioned clinical and pathologic characteristics as well as the absence of data regarding use of endocrine therapy would impair interpretation of the results. Ultimately, the surgical decision making process for each individual patient is complicated. This underscores the importance for each patient to have the opportunity to engage in a multidisciplinary, preoperative discussion to help patients make the most informed decision possible.

In summary, we found that mastectomy rates decreased modestly in elderly women with stage I ER+ breast cancer since publication of the landmark CALGB 9343. The trend in decreased mastectomy rates is mostly driven by elderly NHW women. Hispanic, NHB and NHAPI women continue to have significantly higher mastectomy rates compared to NHW women, even after publication of CALGB 9343. Further study is warranted to explore reasons for the continued high mastectomy rates in elderly, low-risk breast cancer.

## Data Availability

The datasets for this study will not be made publicly available because the data that support the findings of this study are available from the SEER Registry, but restrictions apply to the availability of these data, which were used under license for the current study, and so are not publicly available.

## Ethics Statement

This study was determined exempt by the Institutional Review Board at The Ohio State University.

## Author Contributions

JB and JW wrote the primary manuscript. JB, JF, and MB extracted the data from the SEER database. JF and JB performed the statistical analysis. All authors reviewed the data analysis, study conclusions, contributed to the manuscript revision, read, and approved the submitted version.

### Conflict of Interest Statement

The authors declare that the research was conducted in the absence of any commercial or financial relationships that could be construed as a potential conflict of interest.
